# Skeletal Surveys in Suspected Non-accidental Trauma: Examining the Yield and Current Clinical Practices at a Regional Referral Center in West Virginia

**DOI:** 10.7759/cureus.46020

**Published:** 2023-09-26

**Authors:** Umer Muhammad, Claire Di Bella, Stephanie Thompson, Sharon Istfan

**Affiliations:** 1 Pediatrics, Charleston Area Medical Center, Charleston, USA; 2 Pediatrics, West Virginia University School of Medicine, Charleston, USA; 3 Institute of Academic Medicine, Charleston Area Medical Center, Charleston, USA; 4 Pediatric Hospital Medicine, Vanderbilt University, Nashville, USA

**Keywords:** radiographic imaging, bone calcification, skeletal surveys, child abuse, non-accidental trauma

## Abstract

Introduction

Non-accidental trauma (NAT) is a leading cause of pediatric injury and death. When NAT is suspected in children under the age of 24 months, the American Academy of Pediatrics (AAP) recommends using skeletal surveys (SS) to identify acute, healing, or old fractures and to repeat the SS approximately two weeks after initial imaging as acute fractures can sometimes not be seen on initial imaging. In this study, we determined the yield of initial and follow-up SS obtained for suspected NAT in children under the age of 24 months at a regional referral hospital.

Methods

We reviewed charts of children younger than 24 months who received SS imaging, due to physical abuse suspicion, at our hospital system between 2017 and 2022. We used convenient sampling to examine all SS occurring at the Charleston Area Medical Center Healthcare System.

Results

A total of 61 of the 126 initial SS showed fractures. Only 9% of children received follow-up SS. Repeat SS performed approximately two weeks after positive initial SS showed signs of healing, including new fractures not reported on the initial scan. Follow-up SS performed within eight weeks after initial negative scans continued to be negative. Lastly, consults from child abuse pediatricians were found to be underutilized as only 48% of patients received consultations.

Conclusion

Follow-up SS and child abuse pediatrician consults were found to be underutilized. Follow-up SS and consulting child abuse specialists should not be overlooked, irrespective of positive or negative initial SS, to provide optimal management of NAT.

## Introduction

Non-accidental trauma (NAT) is a leading worldwide cause of preventable morbidity and mortality in young children. The World Health Organization has labeled child maltreatment as an international priority and created a global plan of action to decrease violence against children [1]. Among the possible NAT-related injuries, skeletal fractures are found in 11%-30% of pediatric NAT victims, with pre-verbal and non-ambulatory children having the highest risk of sustaining fractures [2,3]. Overall, 25%-56% of fractures in children less than one year of age can be attributed to physical abuse [4]. The presence of multiple fractures strongly correlates with NAT, especially when fractures exhibit various stages of healing [2]. Therefore, the American Academy of Pediatrics (AAP) subsection on Child Abuse and Neglect recommends obtaining a skeletal survey(s) (SS), a standardized series of whole body radiographic images, for children less than 24 months of age suspected to be NAT victims [5].

Due to delayed bone calcification and healing, follow-up SS can identify fractures not apparent on initial SS, date injuries, and clarify questionable findings. The medical literature supports the value of repeating SS imaging in cases of suspected NAT [6-11] and the AAP recommends follow-up SS approximately two weeks after initial imaging to detect fractures not visible on the initial SS [5]. Bennett et al. examined children with suspected NAT with follow-up SS during an extended time period of 56 days post-initial negative SS imaging [9]. Their extended time period for follow-up surveys was based on the existing literature and x-ray imaging suggesting fracture healing can last up to 56 days. The authors found that repeating SS within the extended time frame produced additional forensically significant findings.

As a result, it is hypothesized that there is additional yield in follow-up SS up to eight weeks after the initial survey. For this study, we examined the yield of initial and follow-up SS imaging obtained for suspected NAT in children under the age of 24 months in West Virginia (WV). The state of WV has one of the United States’ highest NAT incidences, with a rate of 17.0 per 1000 children compared to the national rate of 8.1 per 1000 [12]. Additionally, in 2021, 24.6 infants born per 1000 in WV were removed from their homes due to maltreatment versus the national average of 7 per 1000 [13]. The opioid crises and the rise in substance abuse are potential contributing factors, and very young children continue to be at increased risk of maltreatment [14]. Findings on SS imagining utilization and yields can be used to improve timely detection, follow-up, and management of NAT.

## Materials and methods

Study design and sampling technique

We performed a cross-sectional, retrospective chart review to evaluate the yield of initial and follow-up SS obtained for suspected NAT in young children at a regional referral hospital. We used convenient sampling to examine all SS occurring at the Charleston Area Medical Center (CAMC) Healthcare System (Charleston, WV) between January 1, 2017, and March 1, 2022, in children under the age of 24 months. We initially utilized internal billing codes to identify SS imaging. Electronic health records (EHRs) were reviewed to confirm study inclusion and obtain subject data. All SS imaging due to NAT suspicion occurred either at a pediatric and women’s health-focused hospital (CAMC Women and Children’s Hospital) containing a Children’s Advocacy Center or at a Level 1 trauma hospital (CAMC General Hospital), both located in Charleston, WV. All aspects of the study were approved by the Charleston Area Medical Center, Institute for Academic Medicine Institutional Review Board (approval number 22-835).

Inclusion and exclusion criteria

Children (under the age of 24 months) were eligible for the study if they had documented suspicion as being NAT due to presenting illness, review of systems, physical exam findings, positive and/or negative studies, or injuries not consistent with history suggesting physical abuse. All obtained SS radiographs complied with the AAP [15] and American College of Radiology standards [16] and consisted of 21 images of the appendicular and axial skeleton. Children receiving SS for reasons unrelated to NAT, such as bone dysplasia or metabolic disease, were excluded from the study.

Data collection

Detailed clinical information was extracted via EHR review for subjects meeting all inclusion criteria. Study data was stored de-identified and managed using the password-protected REDCap electronic data capture tool hosted at our institution [17]. The primary outcome of interest was a positive SS defined as imaging significant for any skeletal fracture, displaced versus non-displaced, reactive bone formation, or periosteal reactions. Secondary outcomes of interest included if a follow-up SS occurred, the number of days between SS scans, findings on follow-up SS, and if the child received a child abuse pediatrician consult during the visit. We captured patient characteristic information including age, sex, race, ethnicity (Hispanic vs. non-Hispanic), known past Child Protective Services (CPS) involvement with the child’s household, and indication for SS imaging.

Statistical analysis

Descriptive statistics, such as means, standard deviations, and percentages, were used to summarize the collected data. If continuous variables were non-parametric, medians and ranges were computed. Categorical variables were reported as frequencies and percentages. IBM SPSS, version 19 (IBM Corp., Armonk, NY) was used for all analyses.

## Results

A total of 141 individual children <24 months of age received SS during the five-year study period, with 15 children excluded due to receiving SS for reasons unrelated to NAT suspicion (nine for skeletal dysplasia, three for metabolic disease, one for failure to thrive, one for dog bite, and one for a brief resolved unexplained event unrelated to abuse), leaving 126 individuals meeting all inclusion criteria. The median age was 5.0 months, with 86% of the children under 12 months of age at the time of initial SS imaging. Majority of subjects were male (58%), with 89% being White, and all were non-Hispanic (Table 1). Known past CPS involvement for the patient’s household was documented for 24% of children.

**Table 1:** Patient characteristics

Characteristic	Frequency (%), N = 126
Age, months	
Median (range)	5.0 (0.3-23.0)
Gender	
Males	73 (58%)
Females	53 (42%)
Race	
Black or African American	19 (15%)
White	112 (89%)
Unknown/not reported	3 (2%)
Ethnicity	
Hispanic	0
Non-Hispanic	126 (100%)
Previous Child Protective Services involvement?	
Yes	30 (24%)
No	49 (39%)
Unknown/not documented	47 (37%)
Indication for skeletal scan/suspected physical abuse	
Cutaneous bruising	23 (18%)
History	115 (91%)
Other imaging modality results	38 (30%)
Patient received a child abuse pediatrician consultation?	
No	65 (52%)
Prior to skeletal survey	33 (26%)
After skeletal survey	28 (22%)
Initial skeletal scan imaging	
Negative/no fractures	65 (52%)
Positive/fracture present	61 (48%)
More than 1 fracture present	8 (6%)
Fracture type	
Skull	36 (29%)
Rib	7 (6%)
Upper extremity	10 (8%)
Lower extremity	13 (10%)
Received repeat skeletal scan imaging?	
No	115 (91%)
Yes	11 (9%)

Indications for obtaining SS and suspected physical abuse included injuries not consistent with the provided history (91%), physical findings such as cutaneous bruising (18%), or other x-ray or CT scan imaging findings suggestive of possible NAT (30%). Of the initial SS conducted, 48% indicated fractures, with the most common type being skull fracture (29% of imaged children), followed by lower extremity (10%), upper extremity (8%), and rib fractures (6%). The consultation rate for child abuse pediatric specialists was 48%, with consultations occurring either before or after the SS imaging.

A total of 11 children received follow-up SS, with three children within the AAP-recommended time period of approximately two weeks post-initial SS, with an additional two SS performed within the extended period of 56 days post-initial SS (Table 2). Of these five patients with a follow-up SS within 56 days, one scan (at day 16) showed new fracture imaging, new periosteal reaction on both femurs, not present on the initial SS. The other four patients did not have new findings on the follow-up SS. Of the six patients having follow-up imaging after 56 days, five SS did not have new findings and one had new acute fractures at the time of repeat SS (at day 190), suggestive of a new NAT event.

**Table 2:** Timing and yield of follow-up skeletal surveys

Timing of repeat skeletal survey imaging	Positive initial skeletal survey	Follow-up skeletal survey		Negative initial skeletal survey	Follow-up skeletal survey	
		New fractures	No new fractures		New fractures	No new fractures
7-21 days	2	1	1	1	0	1
22-56 days	2	0	2	0	0	0
>56 days	3	0	3	3	1	2

## Discussion

We investigated the yield of initial and follow-up SS obtained for NAT suspicion in young children. In our study, approximately half of the initial surveys showed fractures; however, less than 10% of subjects received follow-up SS. Our rate was lower than expected, but was similar to that reported by Singh et al. in the largest study to date examining follow-up SS [10]. As any undiagnosed NAT case places children at an increased risk of continued abuse and future injuries, recommendations for follow-up SS should be followed.

No specific fracture type is conclusively indicative of NAT; however, knowledge of fracture patterns specific to age or development level can assist healthcare providers in distinguishing NAT from accidental injuries [5]. Multiple fractures at various stages of healing and rib fractures in young children have the strongest associations with NAT [5,18]. We found rib fractures present in 7 of the 126 imaged children. However, rib fractures sustained due to physical abuse may be easily missed initially due to limited visualization on x-ray imaging [5,18-19]. To some extent, accidental humeral fractures can be differentiated from non-accidental fractures based on location and type, i.e., supracondylar versus spiral, oblique, or mid-shaft/proximal [5,20]. In general, long bone metaphyseal fractures are suggestive of NAT, especially in young and non-ambulatory children [21]. However, relying only on highly suggestive findings of NAT, such as multiple fractures, rib fractures, and long bone metaphyseal fractures, can be misleading, and our study also showed that NAT commonly presented with the findings of bruising and skull fractures. Thus, physicians should include NAT as a possible differential diagnosis when caring for these young, non-ambulatory, non-verbal patients.

Comprehensive NAT diagnosis practices, including initial and follow-up SS, are imperative. In children older than two years, studies report that follow-up SS identify additional clinically relevant information in 14%-61% children having positive findings on initial SS [6-8,10-11]. In our study, one of the five follow-up SS obtained within eight weeks of the initial had new findings. Additionally, follow-up SS can be beneficial in NAT identification when initial SS were negative. Studies report that new, forensically important findings were present in 9%-12% follow-up SS after negative initial SS [8-10]. Consequently, suboptimal rates of follow-up SS are concerning. Possible reasons for poor compliance may include a lack of provider knowledge of the need for follow-up SS. Also, monitoring appropriate follow-up care is often difficult after discharge since primary care providers may be unfamiliar with best practice guidelines, and imaging findings may not be adequately communicated among outlying facilities, treating hospitals, and Children’s Advocacy Center. A limitation of the study was the inability to obtain outlying facility and primary care electronic health records for study patients.

Consistent international guidelines for the diagnostic workup for suspected NAT are lacking, and discrepancies may hinder optimal diagnostic and management practices. In a systematic review of 20 different NAT guidelines across 15 high-income countries, SS imaging was listed in all 20 guidelines but was not systematically recommended [22]. Furthermore, follow-up SS was only mentioned in 14 guidelines, with proposed timing ranging between one and three weeks post-initial SS. Clear and standardized international diagnostic guidelines may further improve NAT diagnosis and management.

To improve imaging practices and care in young suspected NAT patients, various quality improvement initiatives have been described in the published literature. Consulting child abuse specialists in the ED versus waiting until inpatient admission has been shown to improve compliance with clinical guidelines [23]. Secondly, utilizing a multidisciplinary team of pediatricians, ED physicians, child abuse specialists, inpatient physicians, radiologists, social services, and CPS workers increased compliance in obtaining follow-up SS [24]. Additionally, a recent literature review by Manan et al. highlighted the need for a multidisciplinary approach in identifying, diagnosing, and treating pediatric NAT due to diversity in patient presentation signs and risk factors [25]. Increased utilization of child abuse pediatrician consultation would likely improve patient care and guideline adherence.

## Conclusions

NAT is frequently encountered by healthcare providers all over the world. The examined geographic region has one of the United States’ highest rates of child maltreatment, with a steady increase in cases over the past few years. Our study reviewed patient records of NAT with us finding suboptimal rates for follow-up SS at our health system, but when performed, we found additional clinically significant information. Children under 24 months of age with suspected NAT should receive follow-up SS after initial SS, irrespective of positive or negative findings on initial SS. The adoption of quality improvement measures to increase follow-up SS rates would likely improve guideline compliance and patient care of this vulnerable patient population.
